# Structure of the NDH-2 – HQNO inhibited complex provides molecular insight into quinone-binding site inhibitors

**DOI:** 10.1016/j.bbabio.2018.03.014

**Published:** 2018-07

**Authors:** Jessica Petri, Yosuke Shimaki, Wanting Jiao, Hannah R. Bridges, Euan R. Russell, Emily J. Parker, David Aragão, Gregory M. Cook, Yoshio Nakatani

**Affiliations:** a Department of Microbiology and Immunology, University of Otago, Dunedin 9054, New Zealand; b Maurice Wilkins Centre for Molecular Biodiscovery, The University of Auckland, Private Bag 92019, Auckland 1042, New Zealand; c Maurice Wilkins Centre for Molecular Biodiscovery, Biomolecular Interaction Centre, University of Canterbury, Christchurch, New Zealand; d Ferrier Research Institute, Victoria University of Wellington, Wellington, New Zealand; e MRC Mitochondrial Biology Unit, Wellcome Trust/MRC Building, Hills Road, Cambridge CB2 0XY, UK; f Australian Synchrotron, 800 Blackburn Road, Clayton, Victoria, VIC3168, Australia

**Keywords:** NDH-2, Enzyme-inhibitor complex structure, Quinolone, Quinolinyl pyrimidine, Bioenergetics, Electron transport chain

## Abstract

Type II NADH:quinone oxidoreductase (NDH-2) is a proposed drug-target of major pathogenic microorganisms such as *Mycobacterium tuberculosis* and *Plasmodium falciparum*. Many NDH-2 inhibitors have been identified, but rational drug development is impeded by the lack of information regarding their mode of action and associated inhibitor-bound NDH-2 structure. We have determined the crystal structure of NDH-2 complexed with a quinolone inhibitor 2-heptyl-4-hydroxyquinoline-*N*-oxide (HQNO). HQNO is nested into the slot-shaped tunnel of the Q-site, in which the quinone-head group is clamped by Q317 and I379 residues, and hydrogen-bonds to FAD. The interaction of HQNO with bacterial NDH-2 is very similar to the native substrate ubiquinone (UQ_1_) interactions in the yeast Ndi1–UQ_1_ complex structure, suggesting a conserved mechanism for quinone binding. Further, the structural analysis provided insight how modifications of quinolone scaffolds improve potency (*e*.*g*. quinolinyl pyrimidine derivatives) and suggests unexplored target space for the rational design of new NDH-2 inhibitors.

## Highlights

• First crystal structure of bacterial NDH-2 inhibited with the quinolone inhibitor HQNO
• HQNO is a competitive inhibitor of quinone and targets the Q-site of NDH-2.
• A conserved quinone binding mechanism among bacterial and yeast NDH-2 suggested
• Rational design of new NDH-2 quinolone scaffold inhibitors suggested

## Graphical abstract

**Figure:**
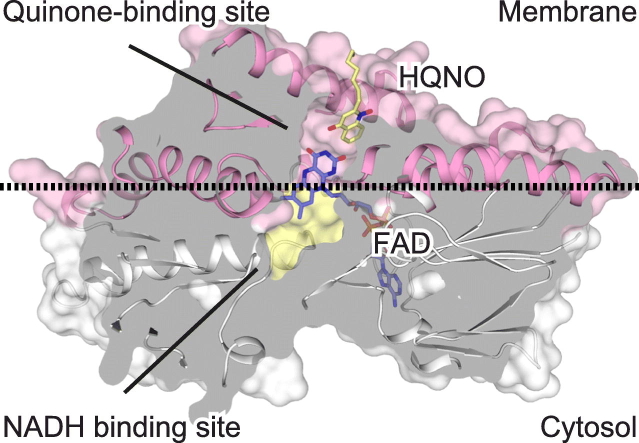

## 1 Introduction

NADH:quinone oxidoreductase is an important enzyme in the respiratory system of many organisms. It serves as a primary entry-point for electrons in the electron transport chain for generation of ATP, and is responsible for maintaining cellular NAD^+^/NADH balance. Unlike the large multi-subunit complex of the proton pumping type I NADH:quinone oxidoreducatase (complex I) [1] and the sodium pumping type NADH:quinone oxidoreducatase (NQR) [2], the type II NADH:quinone oxidoreducatase (NDH-2) is a single subunit monotopic membrane-protein with a molecular mass range of 40–70 kDa [3,4]. NDH-2 catalyses exergonic oxidation of NADH and quinone reduction through the co-factor FAD or FMN [[5], [6], [7]]. These two-half reactions proceed through an atypical ternary mechanism regardless of the presence of the other substrate [6]. Crystal structures of four NDH-2 homologues from *Saccharomyces cerevisiae* [7], *Caldalkalibacillus thermarum* [8], *Staphylococcus aureus* [5], and *Plasmodium falciparum* [9] have been reported. NDH-2 comprises two Rossmann folds that are responsible for binding NADH and housing the co-factor FAD (non-covalently), are central to NADH oxidation. These domains are followed by a C-terminal membrane-anchoring domain in which the quinone-binding site (Q-site) is localised, allowing electron transfer from the reduced FAD to the acceptor quinone pooled in the membrane. In contrast to a canonical nucleotide-binding domain, the Q-site is less conserved among NDH-2 species and structural knowledge regarding how the quinone-substrate binds is very limited with only the yeast Ndi1-ubiquinone (UQ_1_) complex structure available.

**Fig. 1:**
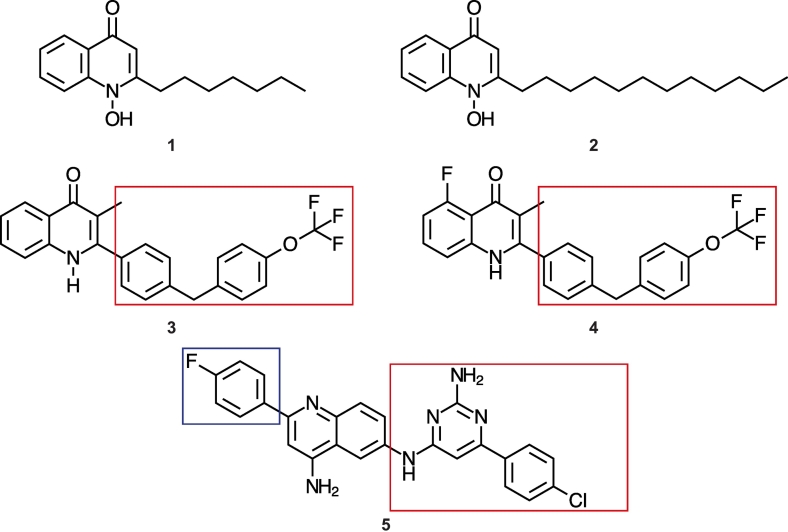
Quinolone and quinolinyl pyrimidine NDH-2 inhibitors described in this study. 1) 2-Heptyl-4-hydroxyquinoline-*N*-oxide. 2) 1-1-Hydroxy-2-dodecyl-4(1*H*)quinolone. 3) Bisaryl quinolone (PfNDH-2 inhibitor) [13] 4) RY-552 (PfNDH-2 inhibitor) [9] 5) Quinolinyl pyrimidine (Mtb NDH-2 inhibitor) [11] Bulky bisaryl and phenyl pyrimidine groups are enclosed in red boxes, and a 4-fluorophenyl group is enclosed in a blue box.

Many organisms, including bacteria, yeast, fungi, plants, and protists, harbour *ndh*-*2* genes in their genomes, but their absence in mammalian genomes makes NDH-2 an attractive target for drug development [4]. In particular, NDH-2 is a highly regarded target for anti-tubercular and anti-protozoal agents [[9], [10], [11], [12], [13]]. This is supported by its essential function in the growth and survival of *Mycobacterium tuberculosis* [14,15] and the parasitic protozoan, *P*. *falciparum*, a causative agent of malaria [16,17]. A number of chemical compounds such as Iodonium derivatives [16,18], flavones [10,19], phenothiazines [10,20], quinolones [5,12,13,17,[21], [22], [23], [24], [25]], quinolinyl pyrimidines [11], nanaomycin A, and polymyxin B [25,26] have been identified as inhibitors of NDH-2. Among these compounds, quinolones, represented by 2-heptyl-4-hydroxyquinoline-*N*-oxide (HQNO) and 1-hydroxy-2-dodecyl-4(1*H*)quinolone (HDQ) (Fig. 1), are the most potent inhibitors of NDH-2 in many species, including *Yarrowia lipolytica* [22], *S*. *cerevisiae* [21], *Gluconobacter oxydans* [25], *T*. *gondii* [24], *P*. *falciparum* [12,13], and *S*. *aureus* [5]. Highly potent derivatives that target NDH-2 have been developed from these scaffolds. For example, Lin et al. noticed that quinolones with longer carbon chains (>C_12_) conferred greater potency (a half-maximal inhibitory concentration (IC_50_) ~ 300 nM) than those with shorter carbon chains (IC_50_ > 2000 nM), against *T*. *gondii Tg*NDH2-I [24]. More recently a structure–activity relationship (SAR) strategy was employed for *P*. *falciparum* NDH-2 (PfNDH-2) inhibitor development, and many quinolone derivatives with low nanomolar affinity and high cellular potency were developed (Fig. 1) [12,13]. A similar approach was adopted for inhibitor development for *M*. *tuberculosis* NDH-2 (Mtb NDH-2), and the quinolone pyrimidine scaffold was discovered to be key feature that conferred higher potency [11]. A number of quinolinyl pyrimidine derivatives with low nanomolar IC_50_ and low micromolar minimum inhibitory concentrations against cell growth have been developed (Fig. 1).

Although many NDH-2 inhibitors have been identified, rational drug design is impeded by the lack of information regarding their modes of action and the unsolved inhibitor-bound structure. Defining the inhibition mechanism of quinolones using conventional inhibition kinetics has proven difficult, and results have suggested both competitive and non-competitive inhibition [21,22,24,25]. Generally, inhibition kinetic investigations of bi-substrate enzymes are challenging, as exemplified by histone acetyltransferase [27,28] and aldehyde dehydrogenase [29]. One needs to define the catalytic mechanism before deciphering the inhibition mechanism, but defining the catalytic mechanism itself is very challenging because bi-substrate catalysis may theoretically follow multiple pathways, such as a random-order ternary complex mechanism, a compulsory-order ternary complex mechanism, or a ping-pong mechanism [27]. This is indeed the case for NDH-2, and its catalytic mechanism has remained unclear until recently [5,6]. In 2017, a crystal structure of the PfNDH-2 complexed with a high-affinity quinolone, RYL-552, was determined [9]. In this structure, the inhibitor unexpectedly bound at the dimer interface and at another site distant from the substrate-binding sites. Thus, the authors suggested an allosteric inhibition mechanism for RYL-552. However, introducing mutations in these sites did not eliminate the inhibition activity of RYL-552, and its inhibition activity remained high with a IC_50_ of 122 nM. This suggests the primary target of RYL-552 might be at the Q-site. Here, we present the crystal structure of the NDH-2–HQNO complex at 2.8 Å resolution. The NDH-2 inhibited structure reveals HQNO bound at the Q-site of NDH-2 and its inhibition mechanism competitive against quinone substrates. Further structural analysis shows a molecular framework for understanding both the binding of physiological quinone substrates and competitive inhibitors for rational drug development targeting NDH-2.

## 2 Materials and methods

### 2.1 Enzyme expression and purification of NDH-2 derivatives

Wild-type (WT) and I379E *C*. *thermarum* NDH-2 derivatives were expressed and purified as described previously [6,8].

### 2.2 NDH-2 inhibitory assay

NADH:menadione oxidoreduction assay was performed at 37 °C in 50 mM Tris-HCl buffer pH 8.0 containing 150 mM NaCl, 1% dimethyl sulfoxide and 1% octylglucoside as previously described [6]. Activity was monitored by following the absorbance change of NADH (340–380 nm, ε = 4.81 mM^−1^ cm^−1^). For the HQNO inhibitory assay final NADH and menadione (MD) substrate concentrations were fixed at 200 and 50 μM, or at 200 and 400 μM, respectively. HQNO concentrations were varied from 0 to 100 μM and 0 to 300 μM for WT and I379E variants respectively to determine IC_50_ values. Enzyme concentrations used were typically 13.5 and 60.0 ng mL^−1^ for the WT, and I379E variants respectively. Each reaction mix was pre-incubated with MD and HQNO for 2 min and the reaction was initiated by adding NADH to the mix. The activity was normalised against a control sample with no HQNO present in the assay mix. Activity assay at each HQNO concentration was performed in triplicate. For the inhibitory assay using a quinolinyl pyrimidine compound final NADH and MD substrate concentrations were fixed at 200 and 50 μM, respectively. Enzyme concentration used was typically 15.0 ng mL^−1^. The compound concentrations tested were 0, 10 and 50 μM, respectively.

### 2.3 Crystallography of the NDH-2–HQNO complex

Crystallisation was performed employing the hanging-drop vapour diffusion method at 18 °C as previously described [30]. NDH-2–HQNO co-crystallisation was carried out using a 0.1 M Bicine–Tris pH 8.5 buffer containing 10% (w/v) PEG 4000, 25% (v/v) ethylene glycol, 75 mM D, l-lysine, 4% (v/v) dimethyl sulfoxide and 1 mM 2-heptyl-4-hydroxyquinoline-*N*-oxide (HQNO). Crystals were harvested on day four, flash-frozen and stored in liquid nitrogen. NDH-2–HQNO co-crystal diffraction data were collected at the Australian Synchrotron MX2 beam-line equipped with an ADSC Quantum 315r detector with 30% beam attenuation, 1 s exposures and 1° oscillation angle. Data were processed using XDS [31]. 176° of data were merged and scaled using Aimless in the CCP4 suite [32], Molecular replacement was performed using Phaser [33] with a WT model (PDB: 5WED). The structures were refined using PHENIX [34] with NCS restraints applied, COOT [35] was used for model building and PyMOL [36] to create the figures.

### 2.4 Molecular modeling of inhibitors into quinone site in PfDNH-2

The structures of RYL552 and Triton X100 were built in Maestro [37] and then prepared for docking using LigPrep [38]. The Q-site in chain A of the PfNDH2 crystal structure (PDB 5JWC) was used for docking. The crystal structure of NDH2 was prepared using Protein Preparation Wizard [39,40]. RYL552 and Triton X100 were then modelled in to the Q-site using Induced Fit docking protocol in Schrödinger Suite [[41], [42], [43]]. The center of grid is defined as the centroid of residues 50, 77, 79, 436, 440, 441, 470, 471, 472, 473, 485, 499, 502, 503, 504, 506, 507 and 601. The van der Waals radii of ligand and receptor atoms were scaled by a factor of 0.5. The 20 best poses of initial docking were kept. Residues within a 5 Å distance of the respective docked ligand, with the exception of residues Q437 and Q441, were refined. The ligands were then re-docked into the receptor using extra precision (XP) mode, to the top 20 newly generated protein structures if the energy was within 30 kcal mol^−1^ of the best-modelled pose.

### 2.5 Accession number for the crystal structure of NDH-2–HQNO complex

Co-ordinates and structure factors have been deposited in the Protein Data Bank with an accession number 6BDO.

## 3 Results and discussion

### 3.1 HQNO targets the Q-site of bacterial *C*. *thermarum* NDH-2 with high specificity and affinity

**Fig. 2:**
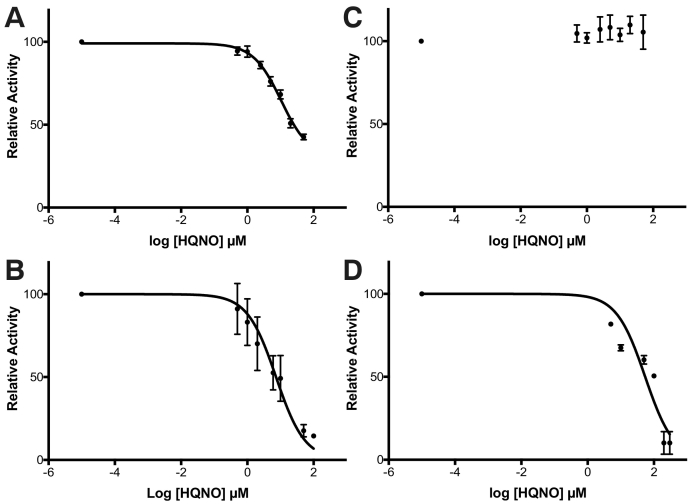
Determination of 2-heptyl-4-hydroxyquinoline- N-oxide (HQNO) IC_50_ for wild-type and I379E NDH-2 variants at two menadione (MD) concentrations. A) and B) HQNO inhibition curves for WT NDH-2 in the presence of 400 and 50 μM MD, respectively. C) and D) HQNO inhibition curves for the I379E NDH-2 mutant in the presence of 400 and 50 μM MD, respectively. Concentration of NADH was set at 200 μM for all experiments. The error bars, which represent 95% confidence intervals, are for triplicate activity assays at each HQNO concentration. A variable slope model was fitted to determine the IC_50_ values. In each panel, NADH:MD oxidoreduction activities in the absence of HQNO are used for 100% and are A) 959.3, B) 562.5, C) 273.4 and D) 82.4 s^−1^, respectively.

NDH-2 is a membrane-bound bi-substrate enzyme that catalyses the cytoplasmic oxidation of NADH and reduction of quinone in the membrane. It is challenging to define the mode of action using conventional enzyme inhibition kinetic methods that rely on obtaining highly accurate rates [21,22,24,25,44]. Instead, we performed a structure-guided inhibition assay using a previously validated I379E *C*. *thermarum* NDH-2 variant, which has significantly reduced quinone-binding affinity (*K*_M_^MD^ 20-fold > wild-type), but retains maximal NADH oxidation activity [6], to evaluate if the NDH-2 inhibitor HQNO specifically targets the Q-site of *C*. *thermarum* NDH-2. We determined the HQNO inhibition activity against the NDH-2 derivatives using menadione (MD) at excess (over ten times the *K*_M_) and low (close to the *K*_M_) concentrations. We expected to observe an inverse correlation between the degree of HQNO inhibition activity and the MD concentration if HQNO and MD directly competed for binding. WT NDH-2 had a IC_50_ value of 10.5 ± 1.3 μM HQNO in the presence of 400 μM MD (Fig. 2A). Because excess MD was present, NDH-2 retained 40% residual activity with 100 μM HQNO. In the presence of 50 μM MD, the IC_50_ value for HQNO decreased slightly to 7.3 ± 1.2 μM and near complete inhibition (~15% residual activity) was observed with >50 μM HQNO (Fig. 2B). Considering both the nearly complete inhibition of NDH-2 activity and the IC_50_ of 7.3 μM in the presence of 50 μM MD, we expected that the HQNO binding affinity will be at least five times that of MD. We estimated the upper limit of the HQNO *K*_D_ was approximately 10 μM using a previously determined *K*_M_^MD^ value of 34 μM [6].

We repeated the same inhibition experiments using a I379E mutant. No inhibition was observed at 100 μM HQNO in the presence of 400 μM MD suggesting I379 has a role in binding HQNO (Fig. 2C). At 50 μM MD, HQNO inhibition was observed with a IC_50_ value of 54.3 ± 1.2 μM (Fig. 2D). Notably, higher HQNO concentrations were required for this mutant to achieve near complete enzyme inhibition at >200 μM. The clear inverse correlation between the MD concentration and the degree of HQNO inhibition activity using the I379E Q-site binding mutant suggests that MD and HQNO compete for binding at this mutated residue. These data are consistent with the observation by Sena et al. that HQNO competitively inhibits binding of a quinone substrate for *S*. *aureus* NDH-2 [5].

### 3.2 NDH-2–HQNO complex structure reveals HQNO specifically bound at the Q-site

**Fig. 3:**
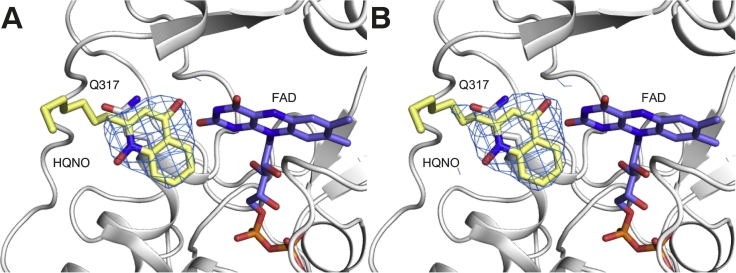
The difference electron density omit map (mFo-Fc) in blue mesh countered at 3.0σ (A) and 2.0σ (B) covers a HQNO molecule (yellow). Packing of the Q317 side chain against the HQNO head group and FAD is represented as a stick model.

To determine the binding of HQNO to the quinone-binding site of NDH-2, we co-crystallised NDH-2 with HQNO using an improved NDH-2 crystallisation platform [30] and determined the complex structure at 2.8 Å resolution (Table 1). The presence of HQNO did not affect the original crystal packing of the NDH-2 enzyme. The structure was solved in the *P*2_1_ space group with lattice parameters that were highly similar to the non-ligand bound and the NAD^+^-bound structures [6,8,30]. After molecular replacement, a distinct electron density that corresponded to the HQNO quinone-head group immediately appeared at the Q-site of two NDH-2 molecules (chains B and C) in an asymmetric unit (Fig. 3). In the two remaining chains, strong peaks were observed at the equivalent sites, but the electron density was slightly disordered. We did not observe any density that might correspond to a second quinone molecule reported in a yeast Ndi1 structure [7], or any other parts of the NDH-2 molecules despite the high concentration of HQNO (1 mM) present in the crystallisation buffer. HQNO molecules were confidently modelled in two chains. Here, we describe the chain B structure, which has lower B-factors for both the protein and ligands than the chain C, as a representative structure.

**Table 1:** Data collection and refinement statistics for the NDH-2–HQNO complex crystal structure.

Wavelength (Å)	0.954
Resolution (outer shell) (Å)	48.0-2.80 (2.89-2.80)
Space group	*P21*
Unit cell parameters	a = 72.9
	b = 114.3
	c = 130.1
	β = 91.2
R_sym_ (outer shell)	0.072 (0.932)
R_pim_ (outer shell)	0.069(0.883)
Mean I/σI (outer shell)	14.5 (1.7)
Completeness (outer shell)	99.8 (100.0)
Multiplicity (outer shell)	3.6 (3.7)
Total No. of reflections	190083 (16726)
No. of unique reflections	52468 (4549)
Mean (I) half-set correlation CC(1/2)	0.998 (0.446)
Wilson B factor (Å^2^)	65.4


Refinement statistics	
Resolution (outer shell) (Å)	43.3-2.80 (2.85-2.80)
R_cryst_	0.227 (0.367)
R_free_	0.268 (0.406)
rmsd for bonds (Å)	0.004
rmsd for angles (deg)	0.838
rmsd for chiral volume (Å^3^)	0.03
No of protein atoms	11603
No. of water atoms	43
No. of FAD atoms	212
No. of HQNO atoms	38
Average main chain *B*-factor (Å^2^)	67.9
Average side chain *B*-factor (Ă^2^)	67.8
Average water *B*-factor (Å^2^)	48.1
Average FAD *B*-factor (Å^2^)	52.7
Average HQNO *B*-factor (Å^2^)	71.2
Ramachandran plot statistics (%)	
Favored regions	97.0
Allowed regions	2.8
Outliers	0.2
PDB entry	6BDO

**Fig. 4:**
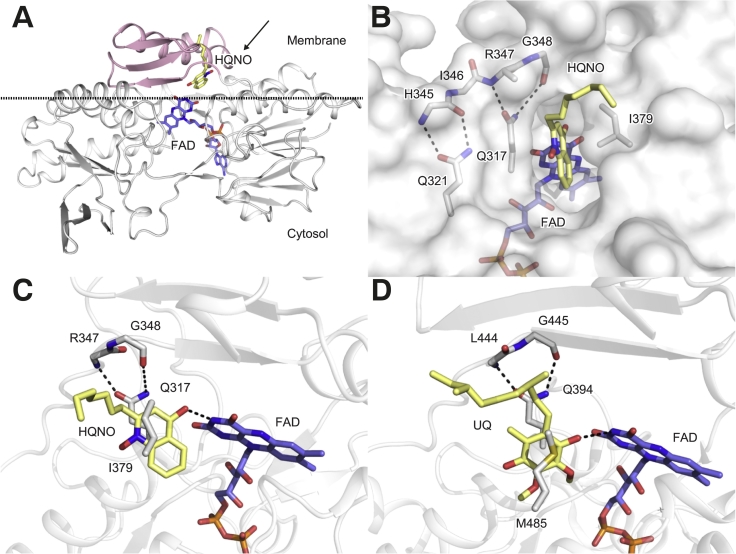
Crystal structure of the NDH-2–HQNO complex. A) An overview of the NDH-2–HQNO complex structure showing the location of the membrane interface (dashed-line), FAD (blue) and HQNO (yellow). The C-terminal membrane anchoring domain is coloured in pink. B) A top view of a panel A (arrowed) showing the quinone-binding site from the membrane side. A pair of Q317 and I379 side chains (white) clamp a quinone-head group of HQNO (yellow). Two conserved glutamate residues (Q317 and Q321) in the AQXAXQ motif holding the linker that separates the NADH- and quinone-binding sites (hydrogen bonds are shown with dashed lines). C) A side view of panel B showing a clamped HQNO head group and a hydrogen bond between the O1 atom of the HQNO quinone head group and the N3 atom of FAD with a distance of 2.8 Å. D) The binding of the quinone head group is conserved for the yeast Ndi1. In the yeast Ndi1–ubiquinone (UQ_1_) complex structure (PDB: 4G73), a UQ (yellow) head group is clamped with Q394 and M485 and also forms a hydrogen bond with FAD (blue).

The structure shows a HQNO molecule bound in the hydrophobic Q-site localising in the C-terminal membrane-anchoring domain, which comprises three anti-parallel β-strands and the first amphipathic helix (Fig. 4A) [8]. The quinone-head group of HQNO is nested into a slot-shaped quinone-binding tunnel with only a few notable contacts established between NDH-2 and HQNO (Fig. 4B). The HQNO aromatic head group is sandwiched by a hydrophobic clamp formed by the pair of Q317 and I379 side chains, consistent with the previous MD docking model [30]. The residue Q317 is present in the highly conserved AQXAXQ motif found in the NDH-2 family proteins [8,45]. Previously, we predicted the structural role of this motif is to hold the linker that separates NADH- and Q-sites exposed to the cytosol and membrane, the side chains of two highly conserved glutamines form tight hydrogen bonds to the backbone of the linker [8,30]. In addition to this structural role, we suggested that the Q317 residue might have a direct role for quinone-binding, given this residue is located immediately next to the *si* face of the FAD isoalloxazine [8]. The NDH-2–HQNO structure provides further evidence that Q317, together with I379, are involved in recognising the quinone (from HQNO) head group. We also noticed only one hydrogen bond, with a distance of 2.8 Å, is formed between a HQNO carbonyl oxygen and a FAD isoalloxazine N3 atom (Fig. 4C). These observations agree with the results of a previous *in silico* quinone-docking study [30]. By contrast, the HQNO alkyl carbon tail is exposed to the solvent and disordered. The positions of the alkyl tail C1 to C3 atoms are supported with weak electron density but the C4 to C7 positions have no supporting electron density (Fig. 3). No other specific interactions, including a hydrogen bond contact between R382 and a ketone oxygen atom of the MD quinone-head that was predicted in an earlier modelling study [30], were observed in the structure. Upon HQNO binding, no major structural changes were observed and the RMSD was 0.41 Å over 394 Cα atoms compared with a non-ligand bound structure (PDB: 5WED). The exception was a D380 side chain carboxyl, which moved away from the Q-site (Fig. S1). Together with previous work that NADH binding does not induce large conformational changes in NDH-2 [6], this new complex structure confirms that ligand-binding in the two substrate-binding sites does not induce conformational changes in bacterial NDH-2.

### 3.3 Comparison of NDH-2–HQNO complex with yeast Ndi1–ubiquinone (UQ_1_) complex

We compared our NDH-2–HQNO complex structure with the yeast Ndi1–UQ_1_ complex structure. Comparison of the two structures identified similar binding orientations of the quinone-head groups in the enzymes (Fig. 4C and D) even though the bacterial respiratory NDH-2 and the eukaryotic yeast inner mitochondrial NDH-2 proteins are evolutionally distant [45], and use different quinone substrates (menaquinone and ubiquinone) [7,8]. A UQ_1_ molecule bound next to a FAD molecule is packed against a conserved glutamine 394 (an equivalent residue to Q317 of *C*. *thermarum* NDH-2). In addition, a hydrophobic side chain of M485 holds the opposite side of the quinone aromatic ring, although M485 is not an equivalent residue to I379 of *C*. *thermarum* NDH-2 (L481 is equivalent). Nonetheless, the packing orientation of the quinone-head groups in both structures was highly comparable (Fig. 4C and D). Another conserved contact found in both structures was a hydrogen bond between a carbonyl oxygen atom of the quinone head group and a N3 atom of the FAD isoalloxazine ring. Although more structural evidence is needed, this conserved quinone-head binding in the two NDH-2 structures might indicate a conserved quinone-binding mechanism in NDH-2. Furthermore, it suggests a common hydride transfer catalytic mechanism mediated *via* a direct hydrogen-bonding interaction between FAD and quinone molecules.

Our bacterial NDH-2–HQNO structure suggests the inhibition mechanism of HQNO is to block the quinone substrate accessing to the FAD isoalloxzine. Furthermore, together with IC_50_ results and the structural similarity in the bound quinone-head group orientation in the bacterial and yeast structures, the mode of HQNO inhibition action against bacterial NDH-2 is competitive against a quinone substrate. In contrast, both competitive and non-competitive inhibition modes of AC0-10 (a HQNO derivative with an extended alkyl tail (C11)) were reported for the yeast Ndi1 [21]. These mixed inhibition modes were dependent on how the catalytic reaction was initiated by adding either NADH or UQ_1_. They could have also arisen from an endogenous UQ_6_ bound in the purified Ndi1 [21] and the second UQ-binding site reported for the Ndi1–UQ_1_ structure [7]. Further experimental evidence is needed to define the HQNO inhibition mechanism for Ndi1. However, structurally, HQNO derivatives could inhibit the activity of Ndi1 by binding at the UQ-binding site (Fig. 4D).

### 3.4 Implications of the HQNO-bound NDH-2 structure to quinolone and quinolinyl pyrimidine NDH-2 inhibitor

**Fig. 5:**
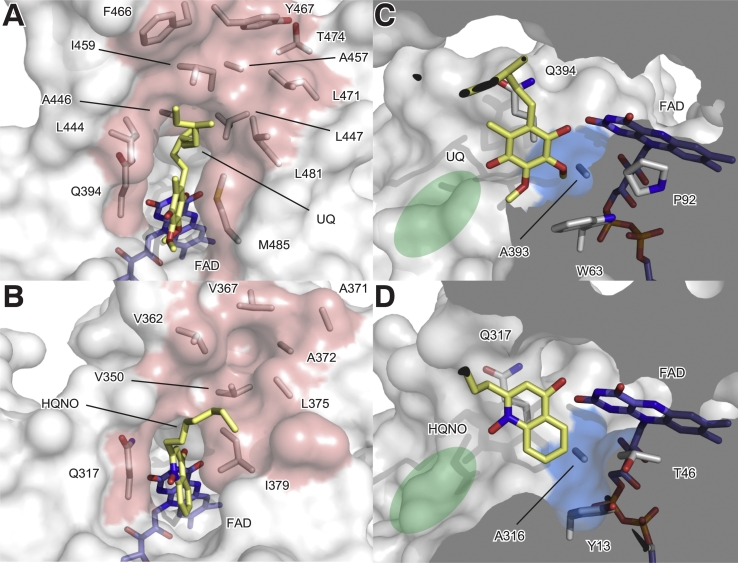
Comparison of the Q-sites of the yeast Ndi1–UQ complex (PDB: 4G73) and the bacterial NDH-2–HQNO complex structure (PDB: 6BDO). A) and B) The hydrophobic groove (light red) formed in the C-terminal anchoring domain of the Ndi1 and the bacterial NDH-2, respectively. C) and D) The hydrophobic cavity (blue) near the FAD molecule (blue stick model) of the Ndi1 and the bacterial NDH-2, respectively. The UQ and HQNO molecules are shown in yellow. The hydrophobic cavities extending away from the quinone-binding tunnel are highlighted in green.

Although a number of quinolone derivatives have been developed, the structural basis for understanding how modifications of quinolone scaffolds improve potency is lacking. Thus, we analysed the NDH-2–HQNO and the Ndi1–UQ structures to gain insight into the molecular basis of the potent inhibition mechanisms of these compounds. In the Ndi1 structure, the ubiquinone isoprenyl tail is accommodated in the hydrophobic groove (L444, L447, I459 and L481) of the C-terminal membrane-anchoring domain (Fig. 5A). In our structure, the HQNO carbon tail extends toward the equivalent hydrophobic groove (V350, V362, L375 and A372), though its tail does not quite reach it because it has a shorter carbon chain (seven carbons) (Fig. 5B). These observations are consistent with the finding that a long quinolone alkyl carbon chain is associated with higher inhibition activity against *T*. *gondii Tg*NDH2-I [24], suggesting the longer carbon chain might be interacting with the hydrophobic groove of the C-terminal domain in *Tg*NDH2-I.

**Fig. 6:**
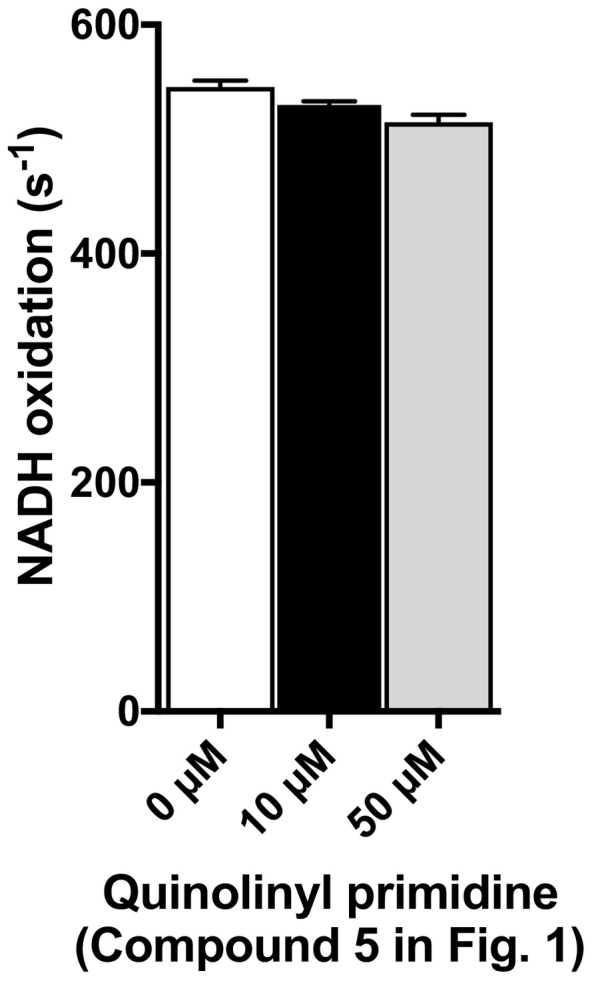
Quinolinyl pyrimidine NDH-2 inhibitor (compound 5 in Fig. 1) does not inhibit bacterial NDH-2. Inhibition activity of the compound 5 was tested against *C*. *thermarum* WT NDH-2 at 0, 10 and 50 μM respectively, in the presence of 50 μM menadione (MD) and 200 μM NADH. Each reaction mix was pre-incubated with MD and the compound 5 for 2 min at 37 °C and the reaction was initiated by addition of NADH. Average values from three technical replicates with error bars ± SEM (n = 3) are bar-graphed. (For interpretation of the references to color in this figure legend, the reader is referred to the web version of this article.)

Highly potent inhibitors for *P*. *falciparum* PfNDH-2 and *M*. *tuberculosis* Mtb NDH-2 have been successfully developed [[11], [12], [13]]. Replacing the alkyl tail of quinolone at the 2-position with an aryl substituent and addition of a methyl group at the 3-position (Fig. 1) led to improved potency against PfNDH-2 [12,13]. Shirude et al. started from the quinolone–pyrimidine scaffold and discovered addition of a phenyl group, both at the 2-position of the quinolone ring and at pyrimidine ring (Fig. 1), improved the potency against Mtb NDH-2 [11]. The conserved orientation of quinone derivatives found in the bacterial and yeast structures might indicate how the chemical modifications improve the potency of these quinolone and quinolinyl pyrimidine derivatives (Fig. 1). Assuming the quinone moieties of quinolone and quinolinyl pyrimidine derivatives bind similarly in PfNDH-2 and Mtb NDH-2, their large bisaryl and phenyl pyrimidine groups most likely target the hydrophobic groove in the C-terminal domain of NDH-2 because of the restricted dihedral angle between the quinone head group and the large hydrophobic moieties. Next, we considered how an additional 4-fluorophenyl group of the quinolinyl pyrimidine derivative (Fig. 1) contributed to improved potency. Structurally, a 4-fluorophenyl group should be accommodated in the hydrophobic area immediately next to FAD (Fig. 5C and D). In the Ndi1 structure, a very shallow hydrophobic patch is formed with W63, P92 and A393, in which one of the UQ methyl ether groups is accommodated (Fig. 5C). Meanwhile, in *C*. *thermarum* NDH-2, the slightly deeper hydrophobic pocket is formed with Y13, T46, and A316 residues (Fig. 5D), suggesting this part might be diverse among NDH-2 species. To accommodate a 4-fluorophenyl group, an equivalent hydrophobic pocket in Mtb NDH-2 must be larger than that of *C*. *thermarum* NDH-2. Supporting this, a quinolinyl pyrimidine compound (Fig. 1) did not show significant inhibition activity against *C*. *thermarum* NDH-2 (Fig. 6). The chemical modifications were focused on these two regions of the quinolone scaffold. To develop a new set of novel NDH-2 inhibitors, the position of the nitrogen oxide can be targeted by medicinal chemistry to add modifications that interact with the hydrophobic area extending away from the quinone-binding tunnel (Fig. 5C and D). As this region of NDH-2 is expected to be diverse [4,7,8,45], the developed inhibitors may indeed have a narrow spectrum of activity.

**Fig. 7:**
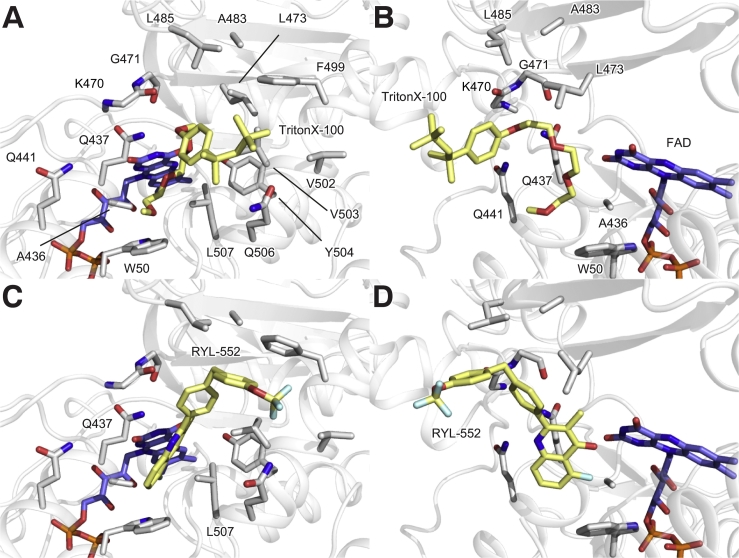
*In silico* docking models of the PfNDH-2–Triton X100 complex and PfNDH-2–RYL552 complex. A) *In silico* docking model of the PfNDH-2–Triton X100 complex. FAD and Triton X-100 molecules are represented as blue and yellow stick models. Residues involved in the hydrophobic groove of the C-terminal membrane anchoring domain are also represented as stick models. B) A view of panel A rotated by 90° clockwise. The first amphipathic helix is omitted for clarity. C) *In silico* docking model of the PfNDH-2–RYL552 complex. FAD and RYL-552 molecules are represented as blue and yellow stick models. Residues involved in the hydrophobic groove of the C-terminal membrane anchoring domain are also represented as stick models. D) A view of panel C rotated by 90° clockwise. The first amphipathic helix is omitted for clarity. Residue labels are omitted for panels C and D as the images are identical to those in panels A and B.

Our analysis suggests that the primary target of the PfNDH-2 inhibitor RYL-552 is the Q-site, given that mutations introduced in the proposed allosteric inhibition sites did not eliminate the inhibition activity of RYL-552 [9]. In addition, in the crystal structure of the PfNDH-2–RYL-552 complex, the disordered electron density at the Q-site, in which Triton X-100 was modelled, was potentially from RYL-552. We tested this hypothesis using the computational ligand-docking simulation. We first docked a Triton X-100 molecule into the Q-site of PfNDH-2 as it was modelled in the crystal structure. We found a similar binding pose to that in the crystal structure *in silico* (Fig. 7A and B), with a docking score of −6.0 kcal mol^−1^. This confirms binding of Triton X-100 in the Q-site is possible. Next, we similarly docked RYL-552, finding that it was comfortably accommodated in the Q-site (Fig. 7C and D) with a lower docking score of −9.5 kcal mol^−1^. This suggests RYL-552 binding is energetically preferred at the Q-site compared with Triton X-100 binding, and the observed disordered density in the Q-site might be from RYL-552. The conserved binding mode of the quinolone head was again predicted in this docking model (Fig. 7C and D). The head group orients immediately next to a FAD isoalloxazine and is clamped by residues Q437 and L507, and a carbonyl oxygen atom of the quinone head group hydrogen bonds to a N3 atom of FAD (with a distance of 2.9 Å). An additional 5-fluoro group binds in a hydrophobic cavity formed with side chains of W50, P79, and A436, and most likely improves the binding affinity. Our *in silico* docking experiment placed a methylene-linked bisaryl with a terminal trifluoromethoxy group into the hydrophobic groove (A483, L485, F499, V502 and V503) of the C-terminal membrane anchoring domain. This result supports our hypothesis that the main inhibition mechanism of the PfNDH-2 inhibitor RY-552 is blocking of the Q-site. Notably, the tetramethylbutyl phenyl group of Triton X-100 contacts the same hydrophobic groove (Fig. 7A and B). When crystallising membrane proteins, a highly-concentrated detergent is unintendedly retained in the protein sample and carried over to the crystallisation drops because the detergent is concentrated during the protein concentration. In fact, a number of Triton X-100 molecules were modelled in the PfNDH-2 structures (PDB: 5JWA, 5JWB, and 5JWC). In addition, a number of unmodelled large disordered electron densities, which are potentially of the detergent, were observed. Similarly, many Triton X-100 have been modelled in yeast Ndi1 structures (PDB: 4G6G, 4G6H, 4G73, and 4G74), and the authors have performed co-crystallisation in the presence of 0.5 mM UQ to obtain Ndi1–UQ complex structures. Therefore, highly-concentrated Triton X-100 and inhibitors might compete for binding at the Q-site of PfNDH-2.

## 4 Conclusions

In conclusion, the structural analysis of bacterial NDH-2 inhibited by HQNO provides molecular details of the interactions and residues involved in quinone binding. The interaction of HQNO with bacterial NDH-2 is very similar to the native substrate UQ interactions in the yeast Ndi1–UQ complex structure suggesting a conserved mechanism for quinone binding, and moreover, a common hydride transfer catalytic mechanism mediated *via* a direct hydrogen-bonding interaction between FAD and quinone molecules among NDH-2 enzymes. The NDH-2–HQNO structure provides insight into the molecular mechanism of inhibition for new NDH-2 inhibitors (*e*.*g*. quinolone and quinolinyl pyrimidine derivatives) and suggests unexplored target space (*e*.*g*. the hydrophobic cavity extending away from the quinone-binding tunnel) for the rational design of new NDH-2 inhibitors. Note that during the peer-review process of this article, another group reported crystal structures of Ndi1 with three Q-site inhibitors (5YJW, 5YJX, and 5YJY) [46].

The following is the supplementary data related to this article.

Fig. S1 The side chain of D380 changes position (cyan to green) upon HQNO binding. The structures of wild-type NDH-2 (wheat) and the HQNO complex (white) are overlaid. Fig. S1

## Author contributions

J.P., Y.S., H.R.B., E.R.R. and Y.N. expressed and purified the protein, and performed assays. D.A. and Y.N crystallised the protein and solved the crystal structure. W.J. performed the *in silico* docking studies. E.J.P., G.M.C. and Y.N. analysed data and wrote the manuscript with help from other authors; E.J.P., G.M.C and Y.N. designed the research and directed the project.

## Competing financial interests

The authors declare no competing interests.

## Transparency document

Transparency document. Image 1
