# Supplementary material for: Structure of the NDH-2 – HQNO inhibited complex provides molecular insight into quinone-binding site inhibitors
Source: Biochim Biophys Acta. 2018 Jul;1859(7):482–90. doi: 10.1016/j.bbabio.2018.03.014 (PMC6167311; doi:10.1016/j.bbabio.2018.03.014)
Supplement: Fig. S1 — The side chain of D380 changes position (cyan to green) upon HQNO binding. The structures of wild-type NDH-2 (wheat) and the HQNO complex (white) are overlaid. [file mmc1.docx]

**Supplementary material**

**Structure of the NDH-2 – HQNO inhibited complex provides molecular insight into quinone-binding site inhibitors**

Jessica Petri^a†^, Yosuke Shimaki^a†^, Wanting Jiao^b,c,d^, Hannah R. Bridges^e^, Euan R. Russell^a^, Emily J. Parker^b,c,d^, David Aragão^f^, Gregory M. Cook^a,b^*, and Yoshio Nakatani^a,b^*

^a^ Department of Microbiology and Immunology, University of Otago, Dunedin 9054, New Zealand

^b^ Maurice Wilkins Centre for Molecular Biodiscovery, The University of Auckland, Private Bag 92019, Auckland 1042, New Zealand

^c^  Maurice Wilkins Centre for Molecular Biodiscovery, Biomolecular Interaction Centre, University of Canterbury, Christchurch, New Zealand

^d^ Ferrier Research Institute, Victoria University of Wellington, Wellington, New Zealand

^e^ MRC Mitochondrial Biology Unit, Wellcome Trust/MRC Building, Hills Road, Cambridge CB2 0XY, UK

^f^ Australian Synchrotron, 800 Blackburn Road, Clayton, Victoria VIC3168, Australia

^†^These authors contributed equally.

*Authors for correspondence: Gregory Cook (gregory.cook@otago.ac.nz) and Yoshio Nakatani (yoshio.nakatani@otago.ac.nz)


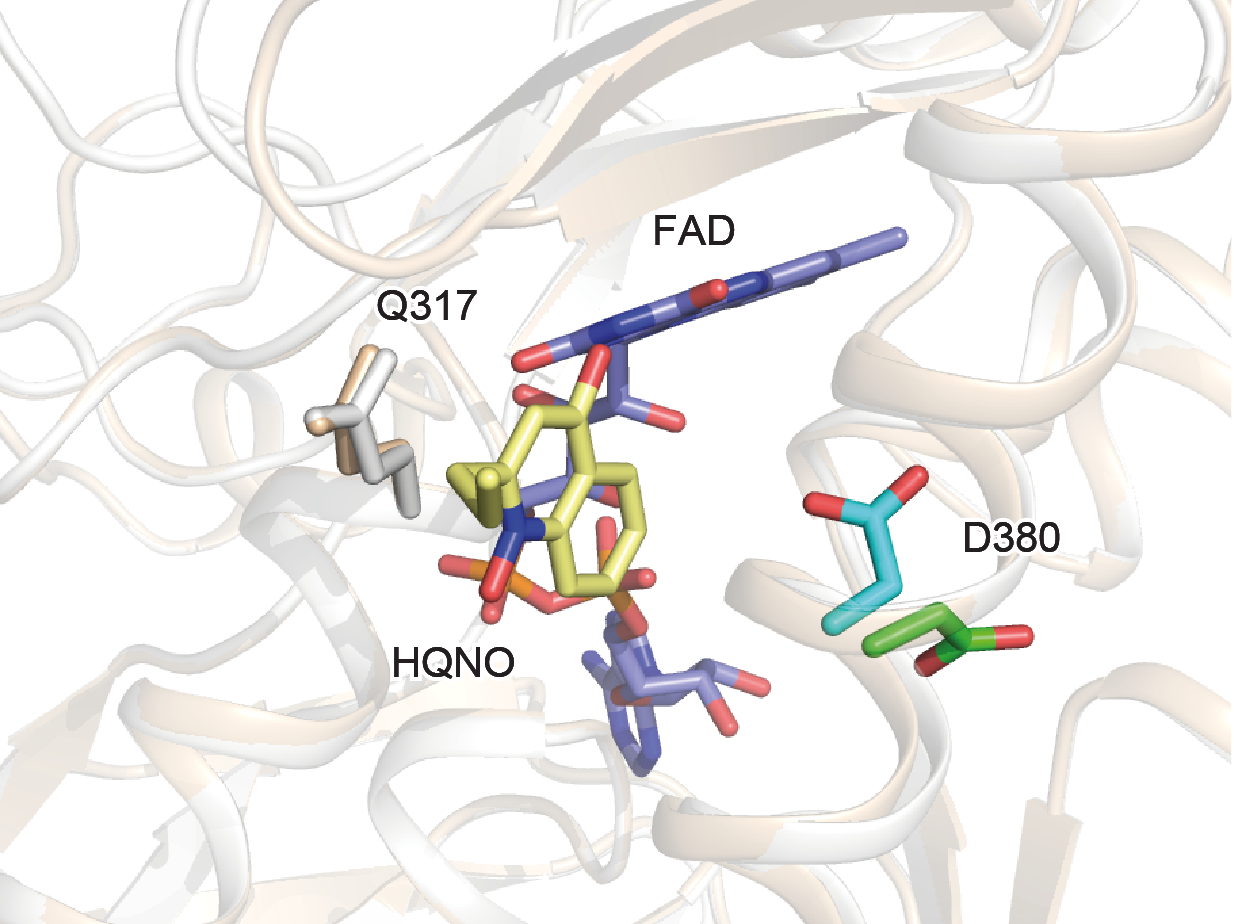


Fig. S1. The side chain of D380 changes position (cyan to green) upon HQNO binding. The structures of wild-type NDH-2 (wheat) and the HQNO complex (white) are overlaid.
